# First report of *Curvularia alcornii* causing leaf spot of quinoa in Bangladesh

**DOI:** 10.1371/journal.pone.0355406

**Published:** 2026-08-04

**Authors:** Amit Hasan, Bilkis Nahar, Bodrun Nessa Shompa, Avizith Kumar Das, M. Salahuddin M. Chowdhury, F. M. Aminuzzaman, Md. Rafiqul Islam, Md. Nazrul Islam, Md. Iyasir Arafat, Sayed Mohammad Mohsin

**Affiliations:** 1 Department of Plant Pathology, Faculty of Agriculture, Sher-e-Bangla Agricultural University, Sher-e-Bangla Nagar, Dhaka, Bangladesh; 2 Department of Genetics and Plant Breeding, Faculty of Agriculture, Sher-e-Bangla Agricultural University, Sher-e-Bangla Nagar, Dhaka, Bangladesh; 3 Plant Biotechnology Division, National Institute of Biotechnology, Ashulia, Savar, Dhaka, Bangladesh; University of Duhok, IRAQ

## Abstract

Quinoa (*Chenopodium quinoa* Willd.) is a nutritionally valuable pseudo-cereal with growing cultivation potential in Bangladesh; however, its productivity is increasingly constrained by foliar diseases, particularly leaf spot. During field surveys at Sher-e-Bangla Agricultural University, Dhaka, quinoa plants exhibiting typical leaf spot symptoms were observed. The present study aimed to identify and characterize the causal agent using an integrated morphological, molecular, phylogenetic, and pathogenicity-based approach. A fungal isolate (SMM-CaQSAU-1) was consistently recovered from infected leaves. Morphological characterization on potato dextrose agar revealed grey to black colonies with abundant aerial mycelia and conidia that were ellipsoidal to curved, dark brown, thick-walled, measuring 18–22 × 8–12 µm. Molecular identification based on BLAST analysis showed that the isolate shared 91.70% sequence similarity in the internal transcribed spacer (ITS) region and 98.39% similarity in the large subunit (LSU) rRNA region with the *Curvularia alcornii* reference strain MFLUCC 10–0703. Phylogenetic analysis using concatenated ITS and LSU sequences placed the isolate within a well-supported *C. alcornii* clade, distinct from other *Curvularia* species. Pathogenicity tests on detached leaves and intact quinoa plants reproduced characteristic leaf spot symptoms within 3–5 days after inoculation, and the pathogen was successfully re-isolated, fulfilling Koch’s postulates. To our knowledge, this study represents the first report in Bangladesh of *C. alcornii* causing leaf spot disease in quinoa. The findings provide critical baseline information for accurate disease diagnosis and highlight a potential emerging threat to quinoa cultivation in Bangladesh, underscoring the need for surveillance, resistance screening, and integrated disease management strategies.

## Introduction

Quinoa (*Chenopodium quinoa* Willd.) is an annual herbaceous pseudo-cereal and facultative halophytic plant that belongs to the Amaranthaceae family [1]. Worldwide, quinoa cultivation is increasing significantly due to its high nutritional value and ability to tolerate harsh climatic conditions [2]. This crop exhibits high genetic diversity, which enhances its adaptability to diverse environments [3]. Quinoa supplies all the essential amino acids in optimum quantities for human life, so it is often recognized as a “superfood” [4]. It contains a higher amount of protein (20%), vitamins, and different types of minerals such as calcium (Ca), magnesium (Mg), and iron (Fe), and is also gluten-free [2]. The glycemic index of quinoa (53) is less than that of white rice, and the calorific content (350 kcal per 100g of grains) is higher than that of many other cereals and legumes [5]. Quinoa seeds can also be used as livestock and poultry feed, along with human consumption. The Food and Agriculture Organization (FAO) has nominated 2013 as the “Year of Quinoa” due to its importance for future food security [2].

Considering this importance, quinoa is also cultivated in some regions of Bangladesh. In Bangladesh, rice, wheat, and maize are the major cereal crops, but these crops are not sufficient to supply all the necessary nutrients. The cultivation of quinoa can contribute to the addition of all essential nutrients. Despite the noticeable rise in demand for quinoa, current supplies remain insufficient to ensure food security.

There are several factors that can reduce quinoa production; in particular, Curvularia leaf spot disease is an important one. Foliage diseases are caused by different fungal species belonging to genera such as *Alternaria, Curvularia, Cercospora, Calonectria, Colletotrichum, Pseudocercospora, Corynespora, Pestalotiopsis, Phyllosticta, Cladosporium, Paramyrothecium, Diaporthe,* etc. [6]. Of these, quinoa leaves are severely infected by the pathogen of *Curvularia* species, which hampers the growth and productivity of quinoa.

*Curvularia* is a dematiaceous filamentous fungus [7]. It is characterized by the brown distoseptate curvature conidia, which are produced from a special structure called a conidiophore. The notable symptoms of *Curvularia* infection are small dot-like chlorosis appearances that show on the leaf at the initial stage, which slowly enlarge into round to oval chlorotic clear spots, including dark brown halos around the edges and yellowish-brown spots in the middle [8]. Apart from their saprophytic nature on the plant surfaces of some species of the fungus, they can parasitize grass-like plants and weeds [8]. *Curvularia* contains a large range of plant hosts, causing immense losses to agricultural sectors by producing various plant diseases [9]. Among *Curvularia* species, *Curvularia alcornii*, a previously unidentified pathogen, was observed causing leaf spot disease in some quinoa plants. Only morphology-based identification of the species of *Curvularia* is incorrect or doubtful, since their morphological characteristics overlap [10]. To overcome this, the present study aimed to identify and characterize the new pathogen affecting quinoa plants in Bangladesh through pathogenicity tests and molecular-based methods, along with morphological analyses. Accurate identification of the disease-causing organism is a crucial step in minimising disease infestation. Although *Curvularia* species are known pathogens causing leaf spot disease, *C. alcornii* has not been previously documented as a causal agent of quinoa leaf spot in Bangladesh. This represents a significant research gap, given the expanding cultivation of quinoa and the potential threat posed by emerging fungal pathogens. Accurate identification and characterization of the pathogen are essential for developing targeted management strategies and safeguarding quinoa production. So, the objectives of our proposed research might be helpful to identify and manage the pathogen that is responsible for causing leaf spot disease of quinoa.

## Materials and methods

### Field inspection and collection of diseased samples

Quinoa leaves, exhibiting typical leaf spot symptoms, were observed in the central field of Sher-e-Bangla Agricultural University. Then the infected leaf samples were excised from the plant and collected into a sterile brown paper envelope. Subsequently, to isolate the pathogen, they were taken to the laboratory of the Department of Plant Pathology at Sher-e-Bangla Agricultural University, Dhaka.

### Isolation, microscopic identification and preservation of the pathogen

Pathogen isolation was conducted by the tissue culture method and designated as SMM-CaQSAU-1. At first, to maintain aseptic conditions, the working bench surface was sterilized with ethanol (70%). Then the infected quinoa leaf samples were cut into small pieces (0.5–1.0 cm) under aseptic conditions and soaked in 1% NaOCl for 30 seconds. Afterwards, with the support of sterile forceps, the sample was carefully picked out and washed 3 times with sterile distilled water to remove the extra NaOCl. The excised infected quinoa leaf samples were kept on sterilized blotter paper (Whatman No. 1) after completing the rinsing process, then cultured on PDA media, followed by incubation at 25 °C for seven days. After incubation, the fungal culture was investigated under a stereo and compound microscope (Motic BA210) to preliminarily identify the pathogen. After identification, purification was done for further study. For preservation, the hyphal tip culture method was used; the pure culture isolate from the PDA was transferred to the PDA slants and stored in a refrigerator at 4 ± 0.5°C for subsequent use.

### Morphological characterization of the pathogen

Morphological characteristics of the pathogen were recorded by transferring the mycelial disc (5 mm) of a 7-day-old culture of the SMM-CaQSAU-1 isolate to the centre of PDA plates. Radial mycelial growth, colony colour, conidia colour, conidia shape, conidia size, and septation of conidia were observed on PDA medium for morphological characterization. Conidia size was measured by using a compound microscope (Motic BA210) and Motic Images Plus 3 software following Mohsin et al. [11].

### Molecular identification of the pathogen

#### Genomic DNA extraction.

Fungal culture was grown on PDA medium overlaid with sterile nitrocellulose membranes for easy hyphal collection. After 10 days, fungal colonies were scraped with an aseptic blade, then it was frozen with liquid nitrogen and finely crushed into powder. The modified Cetyltrimethylammonium Bromide (CTAB) method was used for Genomic DNA extraction [12]. The powdered sample was mixed with preheated CTAB buffer (5 mL per 1 g sample) and 10 μL of mercaptoethanol, then kept for incubation at 65 °C for 40 minutes, and treated with Proteinase K. After CTAB lysis phenol, chloroform and isoamyl alcohol (25:25:1) was added for removal of lipid, protein and others unwanted molecules; followed by centrifugation was done for 15 minutes at 4000 rpm. Then, the aqueous phase from that centrifugation was treated with RNase at 37°C for 30 minutes, precipitated with ice-cold isopropanol overnight, and again centrifuged at 4000 rpm for 20 minutes. This time, the DNA pellet was collected and washed with 70% ethanol; subsequently, TE buffer was used to dissolve it. DNA quality and concentration were assessed using a Nano Drop Spectrophotometer (Thermo Fisher Scientific, USA), and DNA integrity was verified by electrophoresis on a 1% agarose gel. Samples were preserved at −20°C.

#### PCR amplification.

A polymerase chain reaction (PCR) was carried out for amplifying the internal transcribed spacers (ITS) and the larger subunit of rRNA (LSU)regions by using ITS1/ITS4 and LSU-F/LSU-R primer pairs, respectively [13]. PCR amplification conditions are listed in Table 1. PCR amplification was carried out in a 25 µl reaction mixture containing 12.5 µl PCR Master Mix, 1.0 µl of each primer, 1.0 µl genomic DNA, and nuclease-free water to the final volume. PCR products were examined by electrophoresis on a 1.5% agarose gel and visualized under UV illumination.

**Table 1:** List of primers and PCR conditions used for the amplification.

Locus/target genes	Primers	Sequence	Tm	PCR conditions
Larger Subunit of rRNA (LSU)	LSU-F	GTACCCGCTGAACTTAAGC	55°C	Initial denaturation at 95°C for 2 min followed by 35 cycles of denaturation (95°C for 1 min), annealing (55°C for 1 min), extension (72°C for 1 min) and final extension at 72°C for 10 min.
	LSU-R	TCCTGAGGGAAACTTCG		
Internal Transcribed Spacers (ITS)	ITS1F	TCCGTAGGTGAACCTGCGG	48°C	Initial denaturation at 95°C for 2 min followed by 35 cycles of denaturation (95°C for 1 min), annealing (48°C for 1 min), extension (72°C for 1 min) and final extension at 72°C for 10 min.
	ITS4R	TCCTCCGCTTATTGATATGC		

#### Sequence processing and phylogenetic analysis.

With the support of the GeneJET PCR purification kit, PCR products were purified and sequenced via the Sanger di-deoxy method. Forward and reverse chromatograms were inspected manually, and low-quality bases at both ends were trimmed before generating consensus sequences using BioEdit software. The consensus sequences of the representative isolate SMM-CaQSAU-1 were analyzed by using the BLAST (BLASTn) tool (https://blast.ncbi.nlm.nih. gov/Blast.cgi) from the NCBI database. Following this, in order to receive the accession numbers, the sequences were submitted to the NCBI GenBank. The phylogenetic tree was constructed based on multilocus sequences using Molecular Evolutionary Genetics Analysis (MEGA) software (Ver. 11) [14]. The ITS and LSU sequences of all isolates and reference strains were first aligned separately using MEGA 11. Ambiguously aligned regions were manually inspected and trimmed in MEGA 11. After obtaining high-quality individual alignments, the ITS and LSU sequences were concatenated into a single dataset using MEGA 11, ensuring consistent sequence order across all isolates. The concatenated dataset was then used for phylogenetic analysis with the Maximum Likelihood method, and bootstrap support was calculated with 1000 replicates to assess branch confidence. The Maximum-Likelihood method was employed to find out ancestral history, and the Tamura-Nei method [15] was carried out to assess evolutionary distances, along with the confidence values estimated using the bootstrap test (1000 replicates) for clade consistency.

### Pathogenicity test of the pathogen

**Fig 1:**
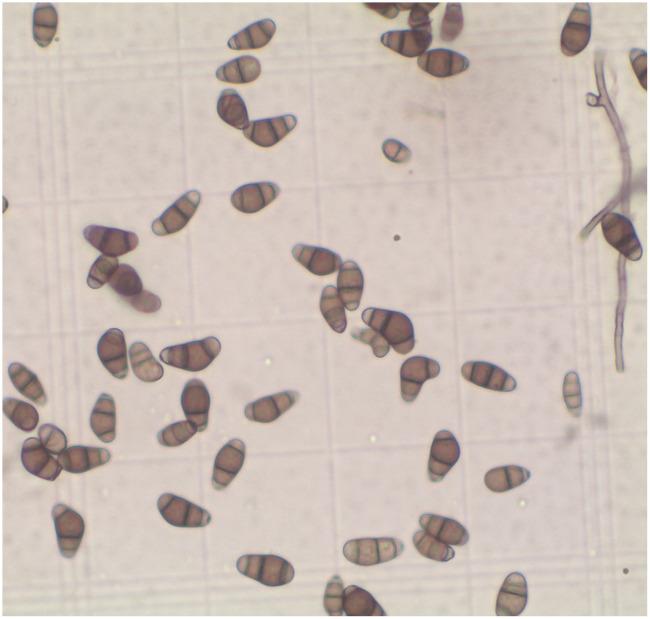
Conidial counting by using a hemocytometer.

For the pathogenicity test, firstly, sterilization of quinoa seeds was maintained by using 70% ethanol, and then the seeds were soaked in distilled water for 10 min. Afterwards, the soaked seeds were sown in a seedling tray containing coco peat and vermicompost (3:1). Five days after sowing, seedlings were transferred into plastic pots (a volume of 2L) to grow hydroponically in the net house. Seedling nourishment was carried out with half-strength Hoagland solution over the growing period, and the nutrient solution was changed and applied every 5 days. At 20 DAS, spore suspension of SMM-CaQSAU-1 isolate was inoculated in two ways, maintaining 1 × 10^8^ conidia mL^–1^, which were counted by a hemocytometer (Fig 1) [11]. In the first procedure, the leaves were detached from plants and placed in a moist plastic growth chamber, then the pathogen was inoculated by creating a wound and subsequently incubated at room temperature for 3 days. In another way, spore suspension was sprayed on the quinoa plants, and humidity was maintained by covering the plants individually with a plastic bag for 5 days. Only sterilized distilled water was sprayed to carry out the control condition. After the appearance of disease symptoms, the confirmation of the leaf spot disease-causing organism was conducted. The experiment was conducted by a completely randomized design (CRD) with three replications, considering homogeneous conditions.

### Statistical analysis

The ITS and LSU sequences of all isolates and reference strains were first aligned separately, and ambiguously aligned regions were manually inspected and trimmed by BioEdit software. After obtaining high-quality individual alignments, the ITS and LSU sequences were concatenated into a single dataset, ensuring consistent sequence order across all isolates. The concatenated dataset was then used for phylogenetic analysis with Maximum Likelihood. Bootstrap support was calculated with 1000 replicates to assess branch confidence. The NCBI-BLAST program (http://blast.ncbi.nlm.nih.gov) was used to analyze the sequence with others. Evolutionary relationship carried out by Molecular Evolutionary Genetics Analysis (MEGA) software (Ver. 11).

## Results

### Symptomology of leaf spot of quinoa

**Fig 2:**
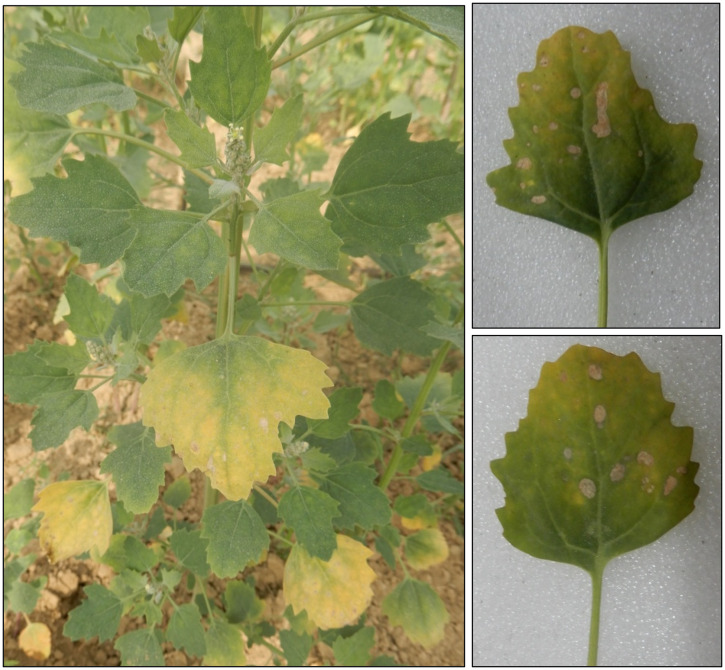
Typical leaf spot symptoms on quinoa caused by *C. alcornii.*

Several small to medium-sized and whitish to light brown spots were observed on quinoa leaves. At the initial stage, small dot-like chlorosis appearances show on the leaves, which slowly enlarge into round to oval whitish to light brown spots. The lesions were spread on the leaf surface with a diameter of 2–6 mm (Fig 2). Lesions appear as small to medium-sized, whitish to light-brown spots, often with dark-brown halos. These symptoms matched the characteristic leaf spot signs reported for *Curvularia* spp. and were consistently observed across infected field samples.

### Isolation and morphological characterization of the pathogen

**Fig 3:**
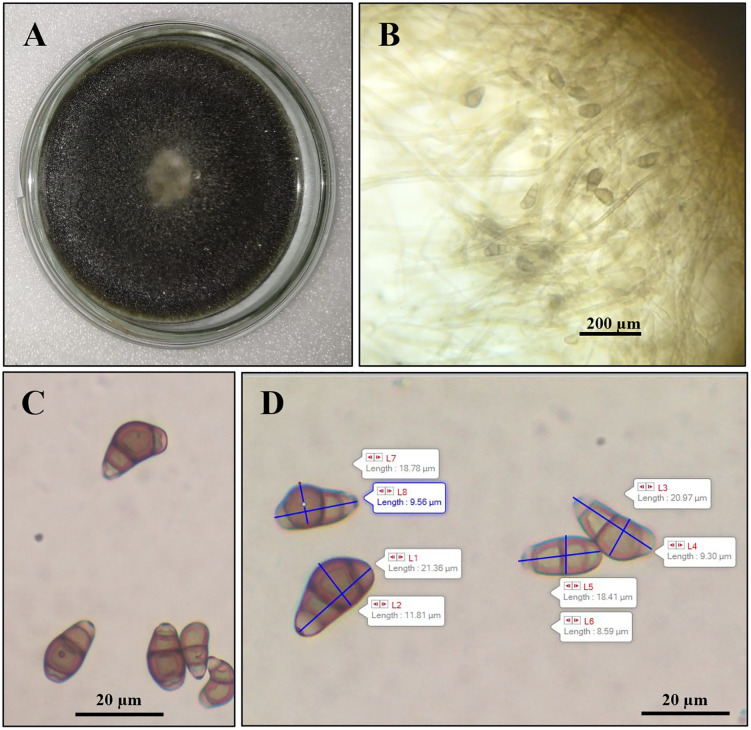
Cultural and morphological characteristics of *C. alcornii* isolate SMM-CaQSAU-1. (A) Pure culture on PDA after 7 days; (B) Mycelium and conidia observed at 100 × magnification; (C) Conidia at 400 × magnification, showing ellipsoidal to curved, dark brown, 1–3-septate morphology; (D) Conidial size measurement (18–22 µm long × 8–12 µm wide).

The fungus isolation takes place on PDA media from the infected quinoa leaves. The fungal colony was observed to be grey to black in color and rich in aerial hyphae; the estimated growth rate was between 5.2 mm per day on PDA media. The conidia were 18–22 μm in length and 8–12 μm in width (Fig 3A-D), produced on the tip of the conidiophore, rounded at both ends, simple, unbranched, thick-walled, straight to curved, ellipsoidal to curved, containing 1–3 septations, where the third cell was larger compared to the others and dark brown in color.

### Molecular characterization of the pathogen

According to the BLAST search, the representative isolate SMM-CaQSAU-1 was identified as *C. alcornii*. The ITS and LSU multilocus DNA sequences obtained from the representative isolate SMM-CaQSAU-1 were submitted to GenBank (http://www.ncbi.nlm.nih.gov), and accession numbers werePQ432867 and PQ432868, respectively, corresponding to each region. Comparison of the SMM-CaQSAU-1 isolate sequences revealed that it is closely related to *C. alcornii*. BLAST analysis showed SMM-CaQSAU-1 shares 91.70% similarity with NR_137091 (ITS) and 98.39% similarity with NG_058842 (LSU), which are sequences from the *C. alcornii* strain MFLUCC 10–0703.

### Phylogenetic analysis

**Fig 4:**
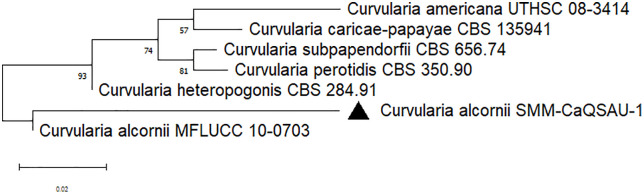
Maximum-likelihood phylogenetic tree based on concatenated ITS and LSU sequences. It shows the relationship between *C. alcornii* SMM-CaQSAU-1 and other *Curvularia* species. Bootstrap values from 1000 replicates are indicated at nodes. The isolate from this study is marked with a black triangle (▲).

The phylogenetic tree constructed by the concatenated sequences of the ITS and LSU genes of SMM-CaQSAU-1 representative isolates shows clustering with the *C. alcornii* strain MFLUCC 10−0703. It was also separate from the other *Curvularia* species, indicating a powerful monophyletic clade (Fig 4). The phylogenetic placement of SMM-CaQSAU-1 within the *C. alcornii* clade, supported by high bootstrap values, confirms its taxonomic assignment. The phylogenetic analysis, which relies on ITS and LSU gene sequences, consistently identifies the fungal pathogen in this study as *C. alcornii*.

### Pathogenicity test to confirm the disease-causing pathogen

**Fig 5:**
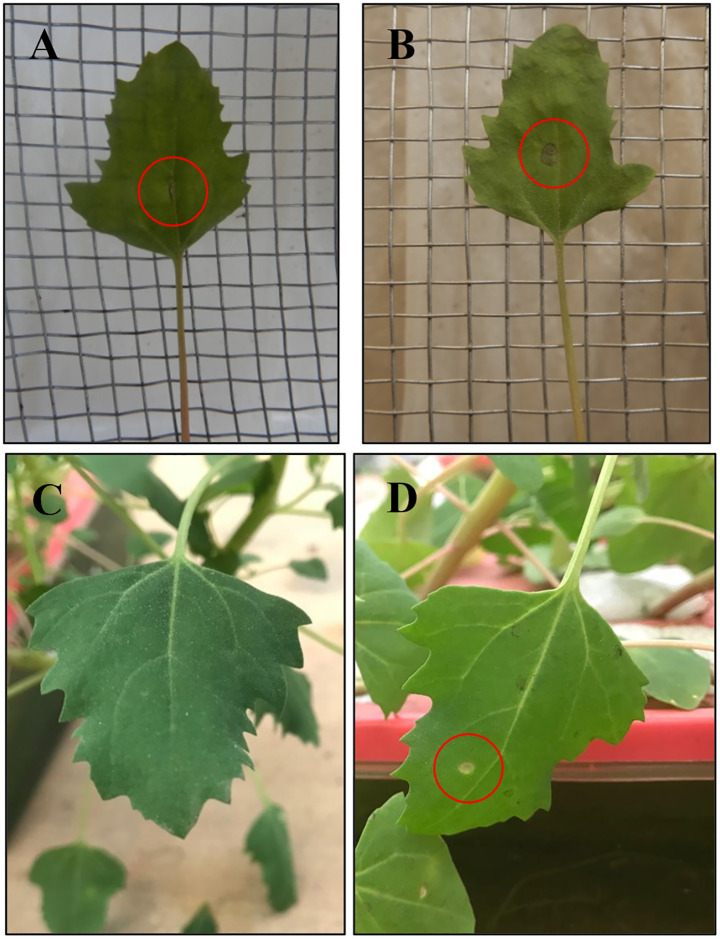
Pathogenicity test of *C. alcornii* SMM-CaQSAU-1 on quinoa. (A) Control detached leaf showing no symptoms; (B) Detached leaf 3 days post-inoculation, showing typical leaf spot lesions; (C) Control intact plant; (D) Quinoa plant leaf 5 days after inoculation, exhibiting characteristic symptoms similar to field observations.

After inoculation of the spore suspension of the isolate SMM-CaQSAU-1, the typical symptoms appeared after three days in detached quinoa leaves and five days in the leaves of quinoa plants (Fig 5A-D). Symptoms on inoculated leaves matched those observed in the field, while control leaves remained asymptomatic. Re-isolation from lesions yielded fungi morphologically identical to the original isolate, fulfilling Koch’s postulates.

## Discussion

Quinoa (*Chenopodium quinoa* Willd.) is a highly nutritious and climate-resilient crop that has gained global importance due to its exceptional nutritional value and tolerance to abiotic stresses. In Bangladesh, quinoa is increasingly recognized as a promising alternative crop for enhancing food and nutritional security. However, like many emerging crops, its production is constrained by diseases, among which foliar leaf spot poses a significant threat. The present study documents *Curvularia* leaf spot of quinoa for the first time in Bangladesh and provides comprehensive evidence identifying the causal agent as *C. alcornii*.

The pathogen associated with the disease was identified using an integrated approach combining morphology, multilocus molecular analysis, phylogenetic inference, and pathogenicity testing. Morphologically, the isolate exhibited characteristic features of *Curvularia*, including dark brown to black, fluffy colonies with abundant aerial mycelium on PDA, and thick-walled, straight to curved, distoseptate conidia with one to three septa and a distinctly enlarged third cell. The morphological characteristics of the present isolate, including colony appearance, conidial shape, septation and dimensions (18–22 × 8–12 µm), were consistent with the published descriptions of *C. alcornii* and closely related species, where described conidia ranged approximately from 17–25 µm in length and 7–13 µm in width [10,16]. This close agreement in size, septation pattern, and conidial morphology provides strong morphological support for the identification. Similar morphological features have been reported from isolates associated with leaf spot diseases in different regions, indicating that the diagnostic trait is relatively stable across geographical locations.

Although morphology is essential for genus-level identification, species delimitation within *Curvularia* based solely on morphological traits is often unreliable due to extensive overlap among closely related species [10,16]. In the present study, molecular analyses revealed comparatively low ITS sequence similarity (91.70%) between isolate SMM-CaQSAU-1 and the reference *C. alcornii* strain MFLUCC 10−0703. In contrast, the LSU region of the isolate showed high similarity (98.39%) with *C. alcornii*, and phylogenetic analysis based on concatenated ITS–LSU sequences placed SMM-CaQSAU-1 within a well-supported monophyletic clade containing authenticated *C. alcornii* strains. The LSU locus has been shown to provide greater phylogenetic stability and deeper taxonomic resolution, particularly when ITS-based identification is ambiguous. Therefore, molecular identification was based on a combined interpretation of ITS and LSU sequence analyses rather than reliance on a single genetic marker. Although the ITS region is widely accepted as the universal fungal barcode, its discriminatory power is sometimes limited within species-rich genera such as *Curvularia*, where closely related taxa may exhibit high sequence similarity or, conversely, considerable intraspecific variation [10]. In the present study, the relatively lower ITS sequence similarity alone was therefore considered insufficient to reject the identification. Instead, the LSU phylogeny provided a more stable taxonomic framework, placing the isolate within the *C. alcornii* lineage with strong bootstrap support.

The pathogenicity test further confirmed the role of *C. alcornii* as the causal agent of quinoa leaf spot, as inoculated plants developed symptoms identical to those observed under field conditions, followed by successful re-isolation of the pathogen. The fulfilment of Koch’s postulates provides definitive biological evidence supporting the etiological role of *C. alcornii*. When interpreted together with the morphological characteristics and successful pathogenicity test, the available evidence consistently supports the identification of the pathogen as *C. alcornii*.

To our knowledge, this is the first report of *C. alcornii* infecting quinoa in Bangladesh. This finding expands the known host range of *C. alcornii* and highlights the adaptive potential of *Curvularia* species to colonize emerging crops. The occurrence of this pathogen on quinoa has important implications for quinoa cultivation in Bangladesh and the broader South Asian region, where warm and humid climatic conditions may favour disease development. Early detection and accurate identification of this pathogen are essential for developing effective disease management strategies, including resistant variety screening, cultural practices, and targeted fungicide application, to ensure sustainable quinoa production.

## Conclusion

The present study provides integrated morphological, molecular, and pathogenicity evidence supporting the identification of *C. alcornii* as the causal agent of leaf spot disease of quinoa in Bangladesh. The findings are consistent with previous reports describing the importance of combining morphological observations with multilocus molecular analyses for accurate identification of *Curvularia* species. Nevertheless, additional studies involving a larger number of isolates from different geographical regions and the inclusion of additional informative loci would further improve our understanding of the genetic diversity, evolutionary relationship, and population structure of this pathogen.
