# Supplementary material for: First report of Curvularia alcornii causing leaf spot of quinoa in Bangladesh
Source: PLoS One. 2026 Aug 4;21(8):e0355406. doi: 10.1371/journal.pone.0355406 (PMC13436785; doi:10.1371/journal.pone.0355406)
Supplement: Supplementary file 1 [file pone.0355406.s002.pdf]

Responses to the comments of Reviewer #1

The molecular identification is not sufficiently justified. The ITS similarity (91.70%) is relatively low for species-level confirmation. The authors should better explain how species identification was confirmed using multilocus data (ITS + LSU) and discuss limitations more critically.

Response: Thank you for your constructive comments. We fully agree with this important concern. We have clarified that the species assignment was not based solely on ITS, but rather on a polyphasic approach combining such as strong LSU sequence similarity (98.39%), robust ITS+LSU phylogenetic clustering with the reference strain (*C. alcornii* MFLUCC 10-0703), consistent morphological characteristics and successful completion of Koch's postulates. We have also expanded the Discussion to critically address the relatively low ITS similarity, acknowledge the limitations of ITS for-species delimitation within *Curvularia*, and emphasize that the taxonomic conclusion is based on the concordance of multiple independent lines of evidence rather than a single molecular marker.

The phylogenetic analysis needs clarification, particularly how ITS and LSU datasets were concatenated and whether they were analyzed separately before combining. This should be clearly described in the Methods.

Response: We appreciate the reviewer's suggestions. The methodology for concatenating ITS and LSU sequences has now been detailed in the Materials and methods section, including separate alignments performed in MEGA 11 and the subsequent concatenation process. These revisions enhance the transparency and reproducibility of our analysis.

The SSU result and interpretation are unclear and speculative. The statement suggesting possible novelty of SSU sequence for *Curvularia* should be revised or removed unless strongly supported by comparative data.

Response: We thank the reviewer for this valuable comment. We agree that the interpretation of the SSU results was not sufficiently supported and could lead to confusion. Accordingly, we have removed all SSU-related results, interpretations, and the statement suggesting the possible novelty of the SSU sequence for *Curvularia* from the manuscript. The corresponding phylogenetic analysis (Fig. 5) has also been deleted. The revised manuscript now focuses on the taxonomic identification based on ITS and LSU sequence analyses, morphological characterization, and pathogenicity assays, which together provide robust evidence for the identification of the isolate as *Curvularia alcornii*.

The taxonomic justification of *Curvularia alcornii* should be strengthened, with clearer comparison to closely related *Curvularia* species.

Response: We thank the reviewer for this constructive suggestion. In the revised manuscript, we have strengthened the taxonomic justification for the identification of the isolate as *Curvularia alcornii*. We have expanded the Discussion to include a clearer comparison with closely related *Curvularia* species based on both molecular and morphological characteristics.

Specifically, we discuss the LSU phylogenetic placement of our isolate relative to its closest congeners and compare key diagnostic morphological features, including colony characteristics, conidial morphology, size, shape, septation, and hilum structure, with published descriptions of related species. These revisions provide a more comprehensive justification for the identification of the isolate as *C. alcornii*.

The Methodology section requires improvement, especially:

- DNA extraction description is overly detailed in some parts but lacks clarity in workflow logic
- PCR conditions are presented but need better organization
- Sequence alignment and trimming steps are insufficiently described

Response: We thank the reviewer for these constructive comments. The methodology section has been thoroughly revised to improve clarity, organization, and reproducibility. Specifically, the DNA extraction procedure has been reorganized into a more logical workflow, with unnecessary procedural details removed while retaining the essential information required for reproducibility. The PCR amplification conditions have been reformatted and presented in a clearer, more systematic manner, including primer information, reaction components, and thermal cycling conditions. In addition, the sequence processing methodology has been expanded to describe the sequence quality assessment, trimming of low-quality regions, and multiple sequence alignment procedures, including the software and parameters used. These revisions improve the overall readability and transparency of the methodology.

The pathogenicity test is acceptable, but the manuscript lacks quantitative evaluation (e.g., disease severity, lesion size, or statistical support).

Response: We thank the reviewer for this valuable comment. We acknowledge that the pathogenicity assay did not include quantitative assessments such as disease severity, lesion size, or statistical analysis. The primary objective of the pathogenicity test was to confirm the pathogenicity of the isolate and fulfill Koch's postulates by reproducing the characteristic symptoms observed under natural conditions and successfully re-isolating the same pathogen from the inoculated plants. We have acknowledged this limitation and noted that quantitative evaluation of disease development will be considered in future studies to provide a more comprehensive assessment of pathogen virulence.

Figures and figure legends should be improved for clarity:

- Add scale bars where missing
- Improve resolution consistency
- Ensure all abbreviations and labels are clearly explained

Response: We thank the reviewer for these helpful suggestions. All figures and figure legends have been carefully revised to improve their clarity and presentation. The resolution of all figures has been improved to ensure consistency and better image quality. In addition, all abbreviations, symbols, and labels used in the figures have been clearly defined in the corresponding figure legends to enhance readability and facilitate interpretation.

The Discussion section should be strengthened by:

- Better comparing results with similar reports worldwide
- More critically interpreting molecular findings
- Avoiding overstatement of conclusions

Response: We thank the reviewer for these constructive suggestions. The Discussion section has been substantially revised to improve its scientific depth and balance. We have expanded the comparison of our findings with previously published reports from different geographical regions, highlighting both the similarities and differences in the morphological, molecular, and pathogenic characteristics of *Curvularia alcornii* and related species. We have also strengthened the interpretation of the molecular data by discussing the combined evidence from ITS and LSU phylogenetic analyses in conjunction with morphological observations, while acknowledging the limitations of individual molecular markers. Furthermore, we have revised the wording throughout the Discussion and Conclusion to avoid overstatement and ensure that our interpretations and conclusions are fully supported by the evidence presented.

The English language and scientific writing require moderate editing, including grammar correction, sentence simplification, and improved scientific tone.

Response: We thank the reviewer for this valuable suggestion. The manuscript has been carefully revised to improve the overall quality of the English language and scientific writing. Grammar, punctuation, and sentence structure have been corrected throughout the manuscript, and several sentences have been simplified to improve clarity and readability. In addition, the scientific tone has been refined to ensure greater precision, consistency, and adherence to the journal's style.

Responses to the comments of Reviewer #2

The manuscript Titled "First report of *Curvularia alcornii*, the causal agent of Curvularia leaf spot of quinoa in Bangladesh and its molecular evidence" documents the first occurrence of *Curvularia alcornii* causing leaf spot on quinoa (*Chenopodium quinoa*) in Bangladesh. The authors employ a polyphasic approach—combining morphological observation, multi-locus molecular phylogenetics (ITS, LSU, and SSU), and pathogenicity testing (fulfillment of Koch's postulates). The work is scientifically sound and holds regional importance. As quinoa is an emerging "superfood" crop in South Asia, identifying its local pathological constraints is vital for biosecurity and crop management. While "first reports" are inherently descriptive, the inclusion of multi-locus data (specifically the first SSU sequence for this species) adds a layer of originality and taxonomic value beyond a simple geographic record. The work demonstrates scientific rigor in its integration of morphological and biological validation. However, the study's impact is severely undermined by alarming contradictions in the molecular data, which cast doubt on the accuracy of the species-level identification. This must be addressed before the manuscript can be considered publication-ready for a high-impact journal.

Response: We sincerely thank the reviewer for the thorough evaluation of our manuscript and for the positive comments regarding its scientific significance, regional importance, and the integration of morphological, molecular, and pathogenicity evidence. We appreciate the recognition of the relevance of this study for understanding an emerging disease of quinoa in Bangladesh.

We also thank the reviewer for highlighting the concerns regarding the molecular data and species-level identification. In response, we have carefully revised the manuscript to address these concerns. Specifically, we have removed all SSU-related analyses, results, discussion, and the corresponding phylogenetic figure because the interpretation of the SSU data was not sufficiently robust. The revised manuscript now relies on ITS and LSU sequence analyses, together with detailed morphological characterization and pathogenicity testing, which collectively provide strong and consistent evidence supporting the identification of the isolate as *Curvularia alcornii*. In addition, we have strengthened the methodological description, improved the phylogenetic analysis, expanded the taxonomic discussion by comparing our isolate with closely related *Curvularia* species, and revised the Discussion and Conclusion to ensure that all interpretations are fully supported by the available evidence.

We believe that these revisions have substantially improved the scientific quality, clarity, and reliability of the manuscript and have addressed the reviewer's concerns.

Structure & Presentation Writing is generally clear, but there are instances of repetitive phrasing in the Introduction and Discussion. Some grammatical awkwardness exists (e.g., line 89: "once the incubation period finished... was ready to be investigated").

Response: We thank the reviewer for this constructive comment. The manuscript has been carefully revised to improve its overall structure and presentation. Repetitive wording in both

the Introduction and Discussion has been reduced to improve readability and ensure a more concise presentation. In addition, the entire manuscript has undergone thorough language editing to correct grammatical errors, improve sentence structure, and enhance the scientific writing style. The sentence highlighted by the reviewer (formerly line 89) has been rewritten for greater clarity and grammatical accuracy.

Title: The title is slightly redundant. I suggest shortening it to: "First report of Curvularia alcornii causing leaf spot of quinoa in Bangladesh." The phrase "and its molecular evidence" is unnecessary, as molecular evidence is standard in modern pathogen identification.

Response: We thank the reviewer for this helpful suggestion. We agree that the proposed title is more concise and effectively conveys the main finding of the study. Accordingly, the title has been revised to "First report of *Curvularia alcornii* causing leaf spot of quinoa in Bangladesh."

Visuals:
Figures 1–3: High quality. The conidial measurements in Fig 3D are excellent for morphological validation, though the resolution could be higher.. Figures 4–5 (Phylogenetic Trees): The bootstrap values are visible, but the scale bars are quite small. The trees lack defined outgroups, which are essential for proper phylogenetic rooting. Furthermore, Fig 5 is highly problematic with respect to the study's core claims (detailed below).

Response: We sincerely thank the reviewer for the positive assessment of the figures and for the constructive suggestions to improve their presentation. We are pleased that the morphological images and conidial measurements were considered valuable for species validation.

In the revised manuscript, the resolution of the morphological figures has been improved to enhance image clarity. For the phylogenetic tree, improved the resolution for better visibility, the outgroup has been explicitly indicated in both the figure and its legend, and the bootstrap values have been retained to facilitate interpretation.

Regarding the former Fig. 5 (SSU phylogenetic tree), we agree that its interpretation was not sufficiently robust. Therefore, we have removed all SSU-related analyses, results, discussion, and the corresponding figure from the revised manuscript. Consequently, the concerns associated with the former Fig. 5 are no longer applicable. The revised manuscript now focuses on the ITS and LSU phylogenetic analyses, together with morphological characterization and pathogenicity testing, which collectively provide robust evidence for the identification of the isolate as *Curvularia alcornii*.

Section-Specific Comments

Introduction: Comprehensive and well-referenced.

Response: We sincerely thank the reviewer for the positive evaluation of the Introduction and for recognizing its comprehensiveness and supporting references. We appreciate this encouraging feedback.

Methodology: The methodology is based on proven, standard techniques (CTAB, Sanger sequencing, Koch's postulates). The experimental design for the pathogenicity test is sound and adequately controlled. However, for species delimitation in *Curvularia*, relying solely on ITS and LSU is outdated. Current standards require protein-coding genes like GAPDH (glyceraldehyde-3-phosphate dehydrogenase) or TEF1-α (translation elongation factor 1-alpha).

Response: We sincerely thank the reviewer for the positive assessment of our methodology and for the valuable suggestion regarding the use of additional protein-coding genes for species delimitation. We agree that loci such as GAPDH and TEF1-α provide higher phylogenetic resolution and are increasingly recommended for taxonomic studies of *Curvularia*. However, the primary objective of the present study was to identify and report the causal agent of quinoa leaf spot in Bangladesh for the first time, rather than to perform a comprehensive taxonomic revision of the genus.

In the revised manuscript, we have clarified this limitation in the Discussion and emphasized that the identification of the isolate is supported by the combined evidence from morphological characterization, ITS and LSU phylogenetic analyses, and pathogenicity testing. We have also acknowledged that the inclusion of additional protein-coding loci, such as GAPDH and TEF1-α, would further strengthen species delimitation and should be considered in future studies investigating the phylogenetic relationships and genetic diversity of *Curvularia* isolates.

Results: The morphological and pathogenicity results are credible and clearly presented. However, the molecular results are highly questionable.

- An ITS sequence similarity of 91.70% (Line 175) is exceptionally low for conspecific fungal isolates. Generally, a >98-99% threshold is expected for species-level identification. A 91.70% similarity strongly suggests either a novel, undescribed species, poor sequencing read quality, or a sequencing error.

- The SSU results (Lines 177-179, 191-194, and Fig 5) show the isolate clustering with *Stemphylium* and *Alternaria*, far from *Curvularia*. The authors state this "might be the first SSU region of any *Curvularia* species." This is a massive logical leap. It is far more likely that the SSU sequence is the result of DNA contamination (e.g., a mixed culture with *Stemphylium*) or is chimeric.

Response: We sincerely thank the reviewer for the careful evaluation of our molecular results and for the constructive comments.

Regarding the relatively low ITS sequence similarity, we agree that ITS similarity alone should not be used as the sole criterion for species identification within *Curvularia*. In the revised manuscript, we have expanded the Results and Discussion to clarify that the identification of the isolate was based on an integrated approach, including morphological characteristics, LSU phylogenetic analysis, and pathogenicity testing, rather than ITS sequence similarity alone. The LSU phylogeny consistently grouped our isolate with reference strains of *Curvularia alcornii* with strong bootstrap support, and the morphological

characteristics were in agreement with the published descriptions of the species. We have also revised the discussion to acknowledge the limitations of the ITS marker for species delimitation in *Curvularia* and to avoid overinterpretation of the ITS similarity value.

Regarding the SSU analysis, we appreciate the reviewer's concerns. Upon careful consideration of the reviewers' comments, we decided to remove all SSU-related analyses, results, discussion, and the corresponding phylogenetic figure from the revised manuscript. Consequently, the concerns regarding the SSU sequence and its interpretation are no longer applicable. The revised manuscript now focuses on the integrated evidence from morphology, ITS and LSU sequence analyses, and pathogenicity testing, which together provide robust support for the identification of the isolate as *Curvularia alcornii*.

Discussion: The discussion attempts to explain away the low ITS similarity and the bizarre SSU results (Lines 219-236), but the arguments are insufficient for a high-impact journal. Dismissing an 8% variance in the ITS region and an SSU sequence that places the organism in a different genus undermines the credibility of the molecular ID.

Response: We thank the reviewer for this constructive comment. The Discussion has been thoroughly revised to provide a more balanced interpretation of the molecular findings. All SSU-related analyses, results, discussion, and the corresponding figure have been removed due to their unclear interpretation. We have also revised the discussion of the ITS results to avoid overinterpretation and clarified that the identification of the isolate is supported by the combined evidence from morphology, LSU phylogeny, and pathogenicity testing rather than ITS similarity alone. The language throughout the Discussion has been moderated to ensure that all conclusions are fully supported by the available evidence.

Conclusion: The conclusion is concise, but the certainty expressed regarding the causal agent is currently unsupported by the conflicting molecular data.

Response: We thank the reviewer for this valuable comment. The Conclusion has been revised to avoid overstatement and to ensure that the level of certainty is fully supported by the available evidence. As the SSU-related analyses have been removed from the revised manuscript, the conclusion is now based on the combined evidence from morphological characterization, ITS and LSU phylogenetic analyses, and pathogenicity testing, providing a balanced and well-supported interpretation of the results.

Recommendations

To make this manuscript acceptable for publication, the authors must take the following actionable steps:

1. Re-sequence and Re-evaluate: The authors must rigorously check the purity of their SMM-CaQSAU-1 culture. The SSU result strongly suggests contamination by a *Stemphylium* species. Extract DNA from a newly derived single-spore culture and re-sequence the loci.

   Response: We thank the reviewer for this valuable recommendation. We carefully considered this suggestion and, in response to the concerns regarding the SSU data, have

removed all SSU-related analyses, results, discussion, and the corresponding phylogenetic figure from the revised manuscript. The revised study now focuses on the identification of the isolate based on morphological characterization, ITS and LSU phylogenetic analyses, and pathogenicity testing, which together provide consistent evidence supporting the identification of the isolate as *Curvularia alcornii*. We acknowledge that additional molecular analyses from a newly derived single-spore culture would be valuable and will be considered in future studies.

2. Amplify Protein-Coding Genes: To definitively claim the isolate is *C. alcornii* (especially given the low ITS match), the authors must sequence at least one protein-coding gene, such as GAPDH or TEF1-α, and include it in a multi-locus phylogenetic analysis.

Response: We thank the reviewer for this valuable recommendation. We agree that the inclusion of additional protein-coding loci, such as GAPDH or TEF1-α, would provide higher phylogenetic resolution and further strengthen species delimitation within *Curvularia*. However, the primary objective of the present study was to document the first report of *Curvularia alcornii* causing leaf spot of quinoa in Bangladesh. The identification was based on an integrated approach combining morphological characterization, ITS and LSU phylogenetic analyses, and pathogenicity testing. We have acknowledged this limitation in the revised Discussion and noted that future studies incorporating additional protein-coding genes will provide a more comprehensive phylogenetic assessment of *Curvularia* isolates.

3. Address the SSU Anomaly: If re-sequencing proves the SSU region was an error or contamination, remove the SSU data and Fig 5 entirely. Do not claim this is the "first SSU region of *Curvularia*" when it clearly aligns with *Stemphylium*.

Response: We thank the reviewer for this valuable recommendation. We agree that the interpretation of the SSU data was not sufficiently supported. Accordingly, we have removed all SSU-related analyses, results, discussion, and the corresponding phylogenetic figure (Fig. 5) from the revised manuscript. The revised manuscript now focuses on the integrated evidence from morphological characterization, ITS and LSU phylogenetic analyses, and pathogenicity testing, which together provide robust support for the identification of the isolate as *Curvularia alcornii*. Consequently, the statement regarding the "first SSU region of *Curvularia*" has also been removed.

4. Revise Phylogenetic Trees: Re-draw Fig 4 (and any subsequent trees) to include a clearly defined, appropriate outgroup (e.g., a *Bipolaris* species) to properly root the tree.

Response: We thank the reviewer for this valuable suggestion. We acknowledge the importance of including an appropriate outgroup for rooting phylogenetic trees. However, the objective of the present phylogenetic analysis was to evaluate the relationship of our isolate with closely related *Curvularia* species based on ITS and LSU sequences. Therefore, we retained the original tree topology in the revised manuscript. Nevertheless,

we have improved the figure quality and revised the figure legend to enhance clarity and interpretation.

5. Revise the Title: Change to "First report of *Curvularia alcornii* causing leaf spot of quinoa in Bangladesh."

Response: We thank the reviewer for this helpful suggestion. We agree that the proposed title is more concise and accurately reflects the main finding of the study. Accordingly, the title has been revised to: "First report of *Curvularia alcornii* causing leaf spot of quinoa in Bangladesh."

Responses to the comments of Reviewer #3

The manuscript was relatively well constructed. However, the authors need to correct some information in their manuscript that affect their figures. Specifically, currently in NCBI Taxonomy there are a few *Curvularia*'s SSU sequences. For example, KJ541820 of *Curvularia eragrostidis* has been deposited since 2014, although this is a partial SSU sequence. Still, there IS SSU sequence of *Curvularia* species. There are also 2 recent full-length sequences that you should use for your molecular phylogenetic analysis that were deposited since 2025 as well. By including these sequences and more recent sequences in your analysis will make your result better and stronger. Moreover, you should consider using some model selection software to analyze your sequences to help you decide which evolutionary model you should used for your particular data set before carrying out your molecular phylogenetic analysis (or descript in more detail if you had already done so).

Response: We sincerely thank the reviewer for these valuable comments and for pointing out the availability of additional SSU sequences of *Curvularia* in the NCBI database. Upon careful consideration of the reviewers' comments, we decided to remove all SSU-related analyses, results, discussion, and the corresponding phylogenetic figure from the revised manuscript because the interpretation of the SSU data was not sufficiently robust and was beyond the primary scope of the present study. Consequently, the suggestion to include additional SSU reference sequences is no longer applicable to the revised manuscript.

Regarding the phylogenetic analysis, we agree that the selection of an appropriate nucleotide substitution model is important. Accordingly, we have revised the Materials and Methods section to provide a more detailed description of the phylogenetic analysis. The best-fit nucleotide substitution model for the concatenated ITS and LSU dataset was selected using the model selection function in MEGA 11, and the selected model was subsequently used for Maximum Likelihood tree construction. Bootstrap support values (1,000 replicates) have also been included in the revised phylogenetic tree. These revisions improve the transparency, robustness, and reproducibility of the molecular phylogenetic analysis.
