# Supplementary material for: First report of Curvularia alcornii causing leaf spot of quinoa in Bangladesh
Source: PLoS One. 2026 Aug 4;21(8):e0355406. doi: 10.1371/journal.pone.0355406 (PMC13436785; doi:10.1371/journal.pone.0355406)
Supplement: Peer review history

# Peer review: Decision Letter 0

**DOI:** 10.1371/journal.pone.0355406.r001

**Authors:** Raed Abduljabbar Haleem

21 Jun 2026

PONE-D-26-24576

First report of Curvularia alcornii, the causal agent of Curvularia leaf spot of quinoa in Bangladesh and its molecular evidence

PLOS One

Dear Dr. Mohsin,

Thank you for submitting your manuscript to PLOS ONE. After careful consideration, we feel that it has merit but does not fully meet PLOS ONE’s publication criteria as it currently stands. Therefore, we invite you to submit a revised version of the manuscript that addresses the points raised during the review process.

Please submit your revised manuscript by Jul 21 2026 11:59PM. If you will need more time than this to complete your revisions, please reply to this message or contact the journal office at plosone@plos.org. When you're ready to submit your revision, log on to https://www.editorialmanager.com/pone/ and select the 'Submissions Needing Revision' folder to locate your manuscript file.

Please include the following items when submitting your revised manuscript:

- A letter that responds to each point raised by the academic editor and reviewer(s). You should upload this letter as a separate file labeled 'Response to Reviewers'.
- A marked-up copy of your manuscript that highlights changes made to the original version. You should upload this as a separate file labeled 'Revised Manuscript with Track Changes'.
- An unmarked version of your revised paper without tracked changes. You should upload this as a separate file labeled 'Manuscript'.

If you would like to make changes to your financial disclosure, please include your updated statement in your cover letter. Guidelines for resubmitting your figure files are available below the reviewer comments at the end of this letter.

If applicable, we recommend that you deposit your laboratory protocols in protocols.io to enhance the reproducibility of your results. Protocols.io assigns your protocol its own identifier (DOI) so that it can be cited independently in the future. For instructions see: https://journals.plos.org/plosone/s/submission-guidelines#loc-laboratory-protocols. Additionally, PLOS ONE offers an option for publishing peer-reviewed Lab Protocol articles, which describe protocols hosted on protocols.io. Read more information on sharing protocols at https://plos.org/protocols?utm_medium=editorial-email&utm_source=authorletters&utm_campaign=protocols.

As the corresponding author, your ORCID iD is verified in the submission system and will appear in the published article. PLOS supports the use of ORCID, and we encourage all coauthors to register for an ORCID iD and use it as well. Please encourage your coauthors to verify their ORCID iD within the submission system before final acceptance, as unverified ORCID iDs will not appear in the published article. *Only*  the individual author can complete the verification step; PLOS staff *cannot*  verify ORCID iDs on behalf of authors.

We look forward to receiving your revised manuscript.

Kind regards,

Raed Abduljabbar Haleem, Ph.D

Academic Editor

PLOS One

Journal Requirements:

When submitting your revision, we need you to address these additional requirements.

1.Please ensure that your manuscript meets PLOS ONE's style requirements, including those for file naming. The PLOS ONE style templates can be found at

https://journals.plos.org/plosone/s/file?id=wjVg/PLOSOne_formatting_sample_main_body.pdf and

https://journals.plos.org/plosone/s/file?id=ba62/PLOSOne_formatting_sample_title_authors_affiliations.pdf

2. Thank you for stating the following in your Competing Interests section:

“The authors declare no competing interests.”

Please complete your Competing Interests on the online submission form to state any Competing Interests. If you have no competing interests, please state "The authors have declared that no competing interests exist.", as detailed online in our guide for authors at http://journals.plos.org/plosone/s/submit-now

This information should be included in your cover letter; we will change the online submission form on your behalf.

3. In the online submission form, you indicated that “Data will be available upon request.”

All PLOS journals now require all data underlying the findings described in their manuscript to be freely available to other researchers, either 1. In a public repository, 2. Within the manuscript itself, or 3. Uploaded as supplementary information.

This policy applies to all data except where public deposition would breach compliance with the protocol approved by your research ethics board. If your data cannot be made publicly available for ethical or legal reasons (e.g., public availability would compromise patient privacy), please explain your reasons on resubmission and your exemption request will be escalated for approval.

4. We notice that your supplementary figures are uploaded with the file type 'Figure'. Please amend the file type to 'Supporting Information'. Please ensure that each Supporting Information file has a legend listed in the manuscript after the references list.

5. If the reviewer comments include a recommendation to cite specific previously published works, please review and evaluate these publications to determine whether they are relevant and should be cited. There is no requirement to cite these works unless the editor has indicated otherwise.

6. Please review your reference list to ensure that it is complete and correct. If you have cited papers that have been retracted, please include the rationale for doing so in the manuscript text, or remove these references and replace them with relevant current references. Any changes to the reference list should be mentioned in the rebuttal letter that accompanies your revised manuscript. If you need to cite a retracted article, indicate the article’s retracted status in the References list and also include a citation and full reference for the retraction notice.

[Note: HTML markup is below. Please do not edit.]

Reviewer's Responses to Questions

**Comments to the Author**

1. Is the manuscript technically sound, and do the data support the conclusions?

The manuscript must describe a technically sound piece of scientific research with data that supports the conclusions. Experiments must have been conducted rigorously, with appropriate controls, replication, and sample sizes. The conclusions must be drawn appropriately based on the data presented.

Reviewer #1: Yes

Reviewer #2: No

Reviewer #3: Partly

**********

2. Has the statistical analysis been performed appropriately and rigorously?

Reviewer #1: Yes

Reviewer #2: N/A

Reviewer #3: No

**********

3. Have the authors made all data underlying the findings in their manuscript fully available?

The PLOS Data policy requires authors to make all data underlying the findings described in their manuscript fully available without restriction, with rare exception (please refer to the Data Availability Statement in the manuscript PDF file). The data should be provided as part of the manuscript or its supporting information, or deposited to a public repository. For example, in addition to summary statistics, the data points behind means, medians and variance measures should be available. If there are restrictions on publicly sharing data—e.g. participant privacy or use of data from a third party—those must be specified.

Reviewer #1: Yes

Reviewer #2: Yes

Reviewer #3: Yes

**********

4. Is the manuscript presented in an intelligible fashion and written in standard English?

PLOS ONE does not copyedit accepted manuscripts, so the language in submitted articles must be clear, correct, and unambiguous. Any typographical or grammatical errors should be corrected at revision, so please note any specific errors here.

Reviewer #1: No

Reviewer #2: Yes

Reviewer #3: Yes

**********

5. Review Comments to the Author

Please use the space provided to explain your answers to the questions above. You may also include additional comments for the author, including concerns about dual publication, research ethics, or publication ethics. (Please upload your review as an attachment if it exceeds 20,000 characters)

Reviewer #1: • The molecular identification is not sufficiently justified. The ITS similarity (91.70%) is relatively low for species-level confirmation. The authors should better explain how species identification was confirmed using multilocus data (ITS + LSU) and discuss limitations more critically.

• The phylogenetic analysis needs clarification, particularly how ITS and LSU datasets were concatenated and whether they were analyzed separately before combining. This should be clearly described in the Methods.

• The SSU result and interpretation are unclear and speculative. The statement suggesting possible novelty of SSU sequence for Curvularia should be revised or removed unless strongly supported by comparative data.

• The taxonomic justification of Curvularia alcornii should be strengthened, with clearer comparison to closely related Curvularia species.

• The Methodology section requires improvement, especially:

o DNA extraction description is overly detailed in some parts but lacks clarity in workflow logic

o PCR conditions are presented but need better organization

o Sequence alignment and trimming steps are insufficiently described

• The pathogenicity test is acceptable, but the manuscript lacks quantitative evaluation (e.g., disease severity, lesion size, or statistical support).

• Figures and figure legends should be improved for clarity:

o Add scale bars where missing

o Improve resolution consistency

o Ensure all abbreviations and labels are clearly explained

• The Discussion section should be strengthened by:

o Better comparing results with similar reports worldwide

o More critically interpreting molecular findings

o Avoiding overstatement of conclusions

• The English language and scientific writing require moderate editing, including grammar correction, sentence simplification, and improved scientific tone.

Reviewer #2: The manuscript Titled “First report of Curvularia alcornii, the causal agent of Curvularia leaf spot of quinoa in Bangladesh and its molecular evidence” documents the first occurrence of Curvularia alcornii causing leaf spot on quinoa (Chenopodium quinoa) in Bangladesh. The authors employ a polyphasic approach—combining morphological observation, multi-locus molecular phylogenetics (ITS, LSU, and SSU), and pathogenicity testing (fulfillment of Koch’s postulates). The work is scientifically sound and holds regional importance. As quinoa is an emerging "superfood" crop in South Asia, identifying its local pathological constraints is vital for biosecurity and crop management. While "first reports" are inherently descriptive, the inclusion of multi-locus data (specifically the first SSU sequence for this species) adds a layer of originality and taxonomic value beyond a simple geographic record.

The work demonstrates scientific rigor in its integration of morphological and biological validation. However, the study's impact is severely undermined by alarming contradictions in the molecular data, which cast doubt on the accuracy of the species-level identification. This must be addressed before the manuscript can be considered publication-ready for a high-impact journal.

Structure & Presentation

Writing is generally clear, but there are instances of repetitive phrasing in the Introduction and Discussion. Some grammatical awkwardness exists (e.g., line 89: "once the incubation period finished... was ready to be investigated").

Title: The title is slightly redundant. I suggest shortening it to: "First report of Curvularia alcornii causing leaf spot of quinoa in Bangladesh." The phrase "and its molecular evidence" is unnecessary, as molecular evidence is standard in modern pathogen identification.

Visuals:

Figures 1–3: High quality. The conidial measurements in Fig 3D are excellent for morphological validation, though the resolution could be higher..

Figures 4–5 (Phylogenetic Trees): The bootstrap values are visible, but the scale bars are quite small. The trees lack defined outgroups, which are essential for proper phylogenetic rooting. Furthermore, Fig 5 is highly problematic with respect to the study's core claims (detailed below).

Section-Specific Comments

Introduction: Comprehensive and well-referenced.

Methodology: The methodology is based on proven, standard techniques (CTAB, Sanger sequencing, Koch's postulates). The experimental design for the pathogenicity test is sound and adequately controlled. However, for species delimitation in Curvularia, relying solely on ITS and LSU is outdated. Current standards require protein-coding genes like GAPDH (glyceraldehyde-3-phosphate dehydrogenase) or TEF1-α (translation elongation factor 1-alpha).

Results: The morphological and pathogenicity results are credible and clearly presented. However, the molecular results are highly questionable.

- An ITS sequence similarity of 91.70% (Line 175) is exceptionally low for conspecific fungal isolates. Generally, a >98-99% threshold is expected for species-level identification. A 91.70% similarity strongly suggests either a novel, undescribed species, poor sequencing read quality, or a sequencing error.

- The SSU results (Lines 177-179, 191-194, and Fig 5) show the isolate clustering with Stemphylium and Alternaria, far from Curvularia. The authors state this "might be the first SSU region of any Curvularia species." This is a massive logical leap. It is far more likely that the SSU sequence is the result of DNA contamination (e.g., a mixed culture with Stemphylium) or is chimeric.

Discussion: The discussion attempts to explain away the low ITS similarity and the bizarre SSU results (Lines 219-236), but the arguments are insufficient for a high-impact journal. Dismissing an 8% variance in the ITS region and an SSU sequence that places the organism in a different genus undermines the credibility of the molecular ID.

Conclusion: The conclusion is concise, but the certainty expressed regarding the causal agent is currently unsupported by the conflicting molecular data.

Recommendations

To make this manuscript acceptable for publication, the authors must take the following actionable steps:

1. Re-sequence and Re-evaluate: The authors must rigorously check the purity of their SMM-CaQSAU-1 culture. The SSU result strongly suggests contamination by a Stemphylium species. Extract DNA from a newly derived single-spore culture and re-sequence the loci.

2. Amplify Protein-Coding Genes: To definitively claim the isolate is C. alcornii (especially given the low ITS match), the authors must sequence at least one protein-coding gene, such as GAPDH or TEF1-α, and include it in a multi-locus phylogenetic analysis.

3. Address the SSU Anomaly: If re-sequencing proves the SSU region was an error or contamination, remove the SSU data and Fig 5 entirely. Do not claim this is the "first SSU region of Curvularia" when it clearly aligns with Stemphylium.

4. Revise Phylogenetic Trees: Re-draw Fig 4 (and any subsequent trees) to include a clearly defined, appropriate outgroup (e.g., a Bipolaris species) to properly root the tree.

5. Revise the Title: Change to "First report of Curvularia alcornii causing leaf spot of quinoa in Bangladesh."

Reviewer #3: The manuscript was relatively well constructed. However, the authors need to correct some information in their manuscript that affect their figures. Specifically, currently in NCBI Taxonomy there are a few Curvularia's SSU sequences. For example, KJ541820 of Curvularia eragrostidis has been deposited since 2014, although this is a partial SSU sequence. Still, there IS SSU sequence of Curvularia species. There are also 2 recent full length sequences that you should use for your molecular phylogenetic analysis that were deposited since 2025 as well.

By including these sequences and more recent sequences in your analysis will make your result better and stronger. Moreover, you should consider using some model selection software to analyze your sequences to help you decide which evolutionary model you should used for your particular data set before carrying out your molecular phylogenetic analysis (or descript in more detail if you had already done so).

**********

6. PLOS authors have the option to publish the peer review history of their article (what does this mean?). If published, this will include your full peer review and any attached files.

If you choose “no”, your identity will remain anonymous but your review may still be made public.

**Do you want your identity to be public for this peer review?**  For information about this choice, including consent withdrawal, please see our Privacy Policy.

Reviewer #1: No

Reviewer #2: **Yes:** Mustafa Adhab

Reviewer #3: No

**********

[NOTE: If reviewer comments were submitted as an attachment file, they will be attached to this email and accessible via the submission site. Please log into your account, locate the manuscript record, and check for the action link "View Attachments". If this link does not appear, there are no attachment files.]

To ensure your figures meet our technical requirements, please review our figure guidelines: https://journals.plos.org/plosone/s/figures

You may also use PLOS’s free figure tool, NAAS, to help you prepare publication quality figures: https://journals.plos.org/plosone/s/figures#loc-tools-for-figure-preparation.

NAAS will assess whether your figures meet our technical requirements by comparing each figure against our figure specifications.

# Author comment: Author response to Decision Letter 1

**DOI:** 10.1371/journal.pone.0355406.r002

13 Jul 2026

Responses to the comments of Reviewer #1

The molecular identification is not sufficiently justified. The ITS similarity (91.70%) is relatively low for species-level confirmation. The authors should better explain how species identification was confirmed using multilocus data (ITS + LSU) and discuss limitations more critically.

Response: Thank you for your constructive comments. We fully agree with this important concern. We have clarified that the species assignment was not based solely on ITS, but rather on a polyphasic approach combining such as strong LSU sequence similarity (98.39%), robust ITS+LSU phylogenetic clustering with the reference strain (C. alcornii MFLUCC 10-0703), consistent morphological characteristics and successful completion of Koch’s postulates. We have also expanded the Discussion to critically address the relatively low ITS similarity, acknowledge the limitations of ITS for-species delimitation within Curvularia, and emphasize that the taxonomic conclusion is based on the concordance of multiple independent lines of evidence rather than a single molecular marker.

The phylogenetic analysis needs clarification, particularly how ITS and LSU datasets were concatenated and whether they were analyzed separately before combining. This should be clearly described in the Methods.

Response: We appreciate the reviewer’s suggestions. The methodology for concatenating ITS and LSU sequences has now been detailed in the Materials and methods section, including separate alignments performed in MEGA 11 and the subsequent concatenation process. These revisions enhance the transparency and reproducibility of our analysis.

The SSU result and interpretation are unclear and speculative. The statement suggesting possible novelty of SSU sequence for Curvularia should be revised or removed unless strongly supported by comparative data.

Response: We thank the reviewer for this valuable comment. We agree that the interpretation of the SSU results was not sufficiently supported and could lead to confusion. Accordingly, we have removed all SSU-related results, interpretations, and the statement suggesting the possible novelty of the SSU sequence for Curvularia from the manuscript. The corresponding phylogenetic analysis (Fig. 5) has also been deleted. The revised manuscript now focuses on the taxonomic identification based on ITS and LSU sequence analyses, morphological characterization, and pathogenicity assays, which together provide robust evidence for the identification of the isolate as Curvularia alcornii.

The taxonomic justification of Curvularia alcornii should be strengthened, with clearer comparison to closely related Curvularia species.

Response: We thank the reviewer for this constructive suggestion. In the revised manuscript, we have strengthened the taxonomic justification for the identification of the isolate as Curvularia alcornii. We have expanded the Discussion to include a clearer comparison with closely related Curvularia species based on both molecular and morphological characteristics. Specifically, we discuss the LSU phylogenetic placement of our isolate relative to its closest congeners and compare key diagnostic morphological features, including colony characteristics, conidial morphology, size, shape, septation, and hilum structure, with published descriptions of related species. These revisions provide a more comprehensive justification for the identification of the isolate as C. alcornii.

The Methodology section requires improvement, especially:

o DNA extraction description is overly detailed in some parts but lacks clarity in workflow logic

o PCR conditions are presented but need better organization

o Sequence alignment and trimming steps are insufficiently described

Response: We thank the reviewer for these constructive comments. The methodology section has been thoroughly revised to improve clarity, organization, and reproducibility. Specifically, the DNA extraction procedure has been reorganized into a more logical workflow, with unnecessary procedural details removed while retaining the essential information required for reproducibility. The PCR amplification conditions have been reformatted and presented in a clearer, more systematic manner, including primer information, reaction components, and thermal cycling conditions. In addition, the sequence processing methodology has been expanded to describe the sequence quality assessment, trimming of low-quality regions, and multiple sequence alignment procedures, including the software and parameters used. These revisions improve the overall readability and transparency of the methodology.

The pathogenicity test is acceptable, but the manuscript lacks quantitative evaluation (e.g., disease severity, lesion size, or statistical support).

Response: We thank the reviewer for this valuable comment. We acknowledge that the pathogenicity assay did not include quantitative assessments such as disease severity, lesion size, or statistical analysis. The primary objective of the pathogenicity test was to confirm the pathogenicity of the isolate and fulfill Koch's postulates by reproducing the characteristic symptoms observed under natural conditions and successfully re-isolating the same pathogen from the inoculated plants. We have acknowledged this limitation and noted that quantitative evaluation of disease development will be considered in future studies to provide a more comprehensive assessment of pathogen virulence.

Figures and figure legends should be improved for clarity:

o Add scale bars where missing

o Improve resolution consistency

o Ensure all abbreviations and labels are clearly explained

Response: We thank the reviewer for these helpful suggestions. All figures and figure legends have been carefully revised to improve their clarity and presentation. The resolution of all figures has been improved to ensure consistency and better image quality. In addition, all abbreviations, symbols, and labels used in the figures have been clearly defined in the corresponding figure legends to enhance readability and facilitate interpretation.

The Discussion section should be strengthened by:

o Better comparing results with similar reports worldwide

o More critically interpreting molecular findings

o Avoiding overstatement of conclusions

Response: We thank the reviewer for these constructive suggestions. The Discussion section has been substantially revised to improve its scientific depth and balance. We have expanded the comparison of our findings with previously published reports from different geographical regions, highlighting both the similarities and differences in the morphological, molecular, and pathogenic characteristics of Curvularia alcornii and related species. We have also strengthened the interpretation of the molecular data by discussing the combined evidence from ITS and LSU phylogenetic analyses in conjunction with morphological observations, while acknowledging the limitations of individual molecular markers. Furthermore, we have revised the wording throughout the Discussion and Conclusion to avoid overstatement and ensure that our interpretations and conclusions are fully supported by the evidence presented.

The English language and scientific writing require moderate editing, including grammar correction, sentence simplification, and improved scientific tone.

Response: We thank the reviewer for this valuable suggestion. The manuscript has been carefully revised to improve the overall quality of the English language and scientific writing. Grammar, punctuation, and sentence structure have been corrected throughout the manuscript, and several sentences have been simplified to improve clarity and readability. In addition, the scientific tone has been refined to ensure greater precision, consistency, and adherence to the journal's style.

Responses to the comments of Reviewer #2

The manuscript Titled “First report of Curvularia alcornii, the causal agent of Curvularia leaf spot of quinoa in Bangladesh and its molecular evidence” documents the first occurrence of Curvularia alcornii causing leaf spot on quinoa (Chenopodium quinoa) in Bangladesh. The authors employ a polyphasic approach—combining morphological observation, multi-locus molecular phylogenetics (ITS, LSU, and SSU), and pathogenicity testing (fulfillment of Koch’s postulates). The work is scientifically sound and holds regional importance. As quinoa is an emerging "superfood" crop in South Asia, identifying its local pathological constraints is vital for biosecurity and crop management. While "first reports" are inherently descriptive, the inclusion of multi-locus data (specifically the first SSU sequence for this species) adds a layer of originality and taxonomic value beyond a simple geographic record.

The work demonstrates scientific rigor in its integration of morphological and biological validation. However, the study's impact is severely undermined by alarming contradictions in the molecular data, which cast doubt on the accuracy of the species-level identification. This must be addressed before the manuscript can be considered publication-ready for a high-impact journal.

Response: We sincerely thank the reviewer for the thorough evaluation of our manuscript and for the positive comments regarding its scientific significance, regional importance, and the integration of morphological, molecular, and pathogenicity evidence. We appreciate the recognition of the relevance of this study for understanding an emerging disease of quinoa in Bangladesh.

We also thank the reviewer for highlighting the concerns regarding the molecular data and species-level identification. In response, we have carefully revised the manuscript to address these concerns. Specifically, we have removed all SSU-related analyses, results, discussion, and the corresponding phylogenetic figure because the interpretation of the SSU data was not sufficiently robust. The revised manuscript now relies on ITS and LSU sequence analyses, together with detailed morphological characterization and pathogenicity testing, which collectively provide strong and consistent evidence supporting the identification of the isolate as Curvularia alcornii. In addition, we have strengthened the methodological description, improved the phylogenetic analysis, expanded the taxonomic discussion by comparing our isolate with closely related Curvularia species, and revised the Discussion and Conclusion to ensure that all interpretations are fully supported by the available evidence.

We believe that these revisions have substantially improved the scientific quality, clarity, and reliability of the manuscript and have addressed the reviewer's concerns.

Structure & Presentation Writing is generally clear, but there are instances of repetitive phrasing in the Introduction and Discussion. Some grammatical awkwardness exists (e.g., line 89: "once the incubation period finished... was ready to be investigated").

Response: We thank the reviewer for this constructive comment. The manuscript has been carefully revised to improve its overall structure and presentation. Repetitive wording in both the Introduction and Discussion has been reduced to improve readability and ensure a more concise presentation. In addition, the entire manuscript has undergone thorough language editing to correct grammatical errors, improve sentence structure, and enhance the scientific writing style. The sentence highlighted by the reviewer (formerly line 89) has been rewritten for greater clarity and grammatical accuracy.

Title: The title is slightly redundant. I suggest shortening it to: "First report of Curvularia alcornii causing leaf spot of quinoa in Bangladesh." The phrase "and its molecular evidence" is unnecessary, as molecular evidence is standard in modern pathogen identification.

Response: We thank the reviewer for this helpful suggestion. We agree that the proposed title is more concise and effectively conveys the main finding of the study. Accordingly, the title has been revised to "First report of Curvularia alcornii causing leaf spot of quinoa in Bangladesh."

Visuals:

Figures 1–3: High quality. The conidial measurements in Fig 3D are excellent for morphological validation, though the resolution could be higher..

Figures 4–5 (Phylogenetic Trees): The bootstrap values are visible, but the scale bars are quite small. The trees lack defined outgroups, which are essential for proper phylogenetic rooting. Furthermore, Fig 5 is highly problematic with respect to the study's core claims (detailed below).

Response: We sincerely thank the reviewer for the positive assessment of the figures and for the constructive suggestions to improve their presentation. We are pleased that the morphological images and conidial measurements were considered valuable for species validation.

In the revised manuscript, the resolution of the morphological figures has been improved to enhance image clarity. For the phylogenetic tree, improved the resolution for better visibility, the outgroup has been explicitly indicated in both the figure and its legend, and the bootstrap values have been retained to facilitate interpretation.

Regarding the former Fig. 5 (SSU phylogenetic tree), we agree that its interpretation was not sufficiently robust. Therefore, we have removed all SSU-related analyses, results, discussion, and the corresponding figure from the revised manuscript. Consequently, the concerns associated with the former Fig. 5 are no longer applicable. The revised manuscript now focuses on the ITS and LSU phylogenetic analyses, together with morphological characterization and pathogenicity testing, which collectively provide robust evidence for the identification of the isolate as Curvularia alcornii.

Section-Specific Comments

Introduction: Comprehensive and well-referenced.

Response: We sincerely thank the reviewer for the positive evaluation of the Introduction and for recognizing its comprehensiveness and supporting references. We appreciate this encouraging feedback.

Methodology: The methodology is based on proven, standard techniques (CTAB, Sanger sequencing, Koch's postulates). The experimental design for the pathogenicity test is sound and adequately controlled. However, for species delimitation in Curvularia, relying solely on ITS and LSU is outdated. Current standards require protein-coding genes like GAPDH (glyceraldehyde-3-phosphate dehydrogenase) or TEF1-α (translation elongation factor 1-alpha).

Response: We sincerely thank the reviewer for the positive assessment of our methodology and for the valuable suggestion regarding the use of additional protein-coding genes for species delimitation. We agree that loci such as GAPDH and TEF1-α provide higher phylogenetic resolution and are increasingly recommended for taxonomic studies of Curvularia. However, the primary objective of the present study was to identify and report the causal agent of quinoa leaf spot in Bangladesh for the first time, rather than to perform a comprehensive taxonomic revision of the genus.

In the revised manuscript, we have clarified this limitation in the Discussion and emphasized that the identification of the isolate is supported by the combined evidence from morphological characterization, ITS and LSU phylogenetic analyses, and pathogenicity testing. We have also acknowledged that the inclusion of additional protein-coding loci, such as GAPDH and TEF1-α, would further strengthen species delimitation and should be considered in future studies investigating the phylogenetic relationships and genetic diversity of Curvularia isolates.

Results: The morphological and pathogenicity results are credible and clearly presented. However, the molecular results are highly questionable.

- An ITS sequence similarity of 91.70% (Line 175) is exceptionally low for conspecific fungal isolates. Generally, a >98-99% threshold is expected for species-level identification. A 91.70% similarity strongly suggests either a novel, undescribed species, poor sequencing read quality, or a sequencing error.

- The SSU results (Lines 177-179, 191-194, and Fig 5) show the isolate clustering with Stemphylium and Alternaria, far from Curvularia. The authors sta

Attachment Submitted filename: Response to Reviewers.pdf

# Editor report: Decision Letter 1

**DOI:** 10.1371/journal.pone.0355406.r003

**Authors:** Raed Abduljabbar Haleem

21 Jul 2026

First report of Curvularia alcornii causing leaf spot of quinoa in Bangladesh

PONE-D-26-24576R1

Dear Dr. Mohsin,

We’re pleased to inform you that your manuscript has been judged scientifically suitable for publication and will be formally accepted for publication once it meets all outstanding technical requirements.

Within one week, you’ll receive an e-mail detailing the required amendments. When these have been addressed, you’ll receive a formal acceptance letter and your manuscript will be scheduled for publication.

An invoice will be generated when your article is formally accepted. Please note, if your institution has a publishing partnership with PLOS and your article meets the relevant criteria, all or part of your publication costs will be covered. Please make sure your user information is up-to-date by logging into Editorial Manager at Editorial Manager® and clicking the ‘Update My Information' link at the top of the page. For questions related to billing, please contact billing support.

If your institution or institutions have a press office, please notify them about your upcoming paper to help maximize its impact. If they’ll be preparing press materials, please inform our press team as soon as possible -- no later than 48 hours after receiving the formal acceptance. Your manuscript will remain under strict press embargo until 2 pm Eastern Time on the date of publication. For more information, please contact onepress@plos.org.

Kind regards,

Raed Abduljabbar Haleem, Ph.D

Academic Editor

PLOS One

Additional Editor Comments (optional):

Reviewers' comments:

# Editor report: Acceptance letter

**DOI:** 10.1371/journal.pone.0355406.r004

**Authors:** Raed Abduljabbar Haleem

PONE-D-26-24576R1

PLOS One

Dear Dr. Mohsin,

I'm pleased to inform you that your manuscript has been deemed suitable for publication in PLOS One. Congratulations! Your manuscript is now being handed over to our production team.

At this stage, our production department will prepare your paper for publication. This includes ensuring the following:

* All references, tables, and figures are properly cited

* All relevant supporting information is included in the manuscript submission,

* There are no issues that prevent the paper from being properly typeset

You will receive further instructions from the production team, including instructions on how to review your proof when it is ready. Please keep in mind that we are working through a large volume of accepted articles, so please give us a few days to review your paper and let you know the next and final steps.

Lastly, if your institution or institutions have a press office, please let them know about your upcoming paper now to help maximize its impact. If they'll be preparing press materials, please inform our press team within the next 48 hours. Your manuscript will remain under strict press embargo until 2 pm Eastern Time on the date of publication. For more information, please contact onepress@plos.org.

You will receive an invoice from PLOS for your publication fee after your manuscript has reached the completed accept phase. If you receive an email requesting payment before acceptance or for any other service, this may be a phishing scheme. Learn how to identify phishing emails and protect your accounts at https://explore.plos.org/phishing.

If we can help with anything else, please email us at customercare@plos.org.

Thank you for submitting your work to PLOS ONE and supporting open access.

Kind regards,

PLOS ONE Editorial Office Staff

on behalf of

Dr. Raed Abduljabbar Haleem

Academic Editor

PLOS One
